# Cognitive Alterations Associated with Remission and Alcohol Dependence Severity in Ethnically Diverse Patients of Siberia

**DOI:** 10.2174/0117450179297171240522051748

**Published:** 2024-06-05

**Authors:** Anastasia Peshkovskaya

**Affiliations:** 1 Tomsk State University, Tomsk, Russia; 2 Mental Health Research Institute, Tomsk National Research Medical Center, Russian Academy of Sciences, Tomsk, Russia

**Keywords:** Alcohol dependence, Cognition, Executive functions, Remission, Relapse, Ethnicity, Indigenous, Intervention, Cognitive training

## Abstract

**Background:**

Impaired cognition in individuals with alcohol dependence may be associated with increased relapse risk. It has been recorded in more than half of patients during six months after treatment. In certain ethnic groups, for example, Tuvinians, the indigenous people of Siberia, relapses occur in extremely short periods of one to three months after treatment. An approach currently used to alcohol dependence treatment may be less effective for these patients.

**Objective:**

The study aimed to investigate cognitive sequelae in indigenous Tuvinian patients with alcohol dependence.

**Methods:**

The sample included 166 patients, 74 of indigenous ethnicity (Tuvinians) and 92 non-indigenous white patients. Data on inhibitory control, cognitive flexibility, attention, and working memory were collected from all the patients and processed using cluster analysis. The clustering data were then complemented by indicators of disorder dynamics, impulsivity, and emotion regulation.

**Results:**

The clustering procedure revealed groups with severe cognitive sequelae. More than four-fold attention decrease was found in 43.5% of non-indigenous patients, and more impaired cognitive flexibility was revealed among 60.8% of indigenous patients. Groups with severe cognitive sequelae had higher impulsivity, maladaptive emotion regulation, more hospitalizations, faster disease progression, and shorter remissions. The latter was significantly reduced to 90 days on average in the severe group of indigenous patients *versus* 135 days of remission in the non-indigenous severe group.

**Conclusion:**

Results obtained may advance tailored intervention in alcohol-dependent patients of the indigenous Tuvinian ethnicity. While little is still known about the alcohol dependence course and consequences in the indigenous Tuvinians of Siberia, this study contributes to the global mental health data on alcohol abuse and dependence in indigenous communities.

## 1 INTRODUCTION

Cognitive sequelae are key manifestations of cerebral damage in alcohol dependence [1-4]. Executive cognition, *i.e*., cognitive control, cognitive flexibility, decision-making, and working memory, are primarily affected by alcohol intoxication [5-9]. With chronic alcohol use, impaired memory and executive cognition are still observed even after one to three weeks of abstinence [10-12]. Recovery of cognitive functions normally occurs after six months of alcohol abstaining; however, impairments in cognitive control and decision-making can persist for up to a year of abstinence [13, 14]. When coupled with impulsive behavior, impaired executive cognition that persists for months after stopping alcohol use can cause patients to relapse to alcohol consumption [15, 16].

Literature showed that impairments in cognitive control, decision-making, and working memory have been widely investigated among factors that increase alcohol dependence relapse risk [17, 18], which is recorded in up to 60% of patients during the first six months after treatment [19, 20]. Importantly, in certain ethnic groups, for example, Tuvinians, the indigenous people of Siberia, an average remission is reduced to an extremely limited period of one to three months after a course of treatment [21, 22]. However, little is known about factors that affect remission in Tuvinian patients.

Tuvinians are continental Mongoloids, one of the indigenous peoples of the Altai-Sayan uplands in the south of Siberia, which is today’s Republic of Tyva, the region of Russia, where Tuvinians make up a predominant population. Their traditional religion is Buddhism with elements of more ancient beliefs, such as shamanism. Cattle breeding has been the primordial main occupation of Tuvinians. For centuries, they brewed a traditional alcoholic drink known as *araga* from boiled, fermented milk. *Araga* was considered a sacred drink prohibited for women, children, and men aged less than 30 years [21]. However, acculturation had an impact on traditional alcohol-related taboos and habits in Tuvinians, and culturally alien pattern and amount of drinking led to much more serious health consequences for them.

Rates of alcohol-related problems among Tuvinians are high. In 2021, 3,339 patients (1,010.7 individuals per 100,000 people) with alcohol use disorders, including alcohol dependence and alcohol-induced psychoses, were registered in the Republic of Tyva [23]. This indicator is 39% higher than the average figure per 100,000 residents of the Siberia region and 23% higher than the overall index in Russia. In addition, remissions in alcohol-dependent Tuvinians are significantly shorter than in the non-indigenous population [21, 22]. Since impaired executive cognition is an important determinant of alcohol dependence as it directly impacts the ability to maintain remission, understanding cognitive alterations in alcohol-dependent patients of indigenous Tuvinian ethnicity, who had extremely short remissions, will advance treatment in this patient group.

### 1.1 Objective

Considering the above, this study aimed to investigate executive cognition in indigenous Tuvinian patients with alcohol dependence.

## 2 METHODS

A minimal sample size of 70 for each subgroup of both indigenous and non-indigenous patients was calculated based on effect size. This study involved 174 men with alcohol dependence according to ICD-10 Code F10.2, including 79 indigenous Tuvinian patients and 95 non-indigenous white Russian patients. All the patients signed an informed consent form to participate in the study and data processing. The criteria for inclusion were alcohol dependence according to ICD-10, a detoxified condition outside alcohol withdrawal and before psychopharma- cological therapy, the age between 25 and 55 years, and consent to participate in the study. Exclusion criteria were comorbid mental conditions, chronic somatic diseases in the acute phase, disorders of the central nervous system, intellectual disability, uncorrected ophthalmic disorders, or refusal to participate in the study. Eight patients did not meet the inclusion criteria and were excluded from the study. The flowchart of the study sample is described in Fig. (**1**).

The final sample consisted of 166 people: 74 indigenous patients and 92 non-indigenous patients who were comparable by age (p = 0.484), education (p = 0.600), and alcohol dependence duration (p = 0.195) based on the Mann–Whitney U test with a continuity correction. Table **1** incorporates the data on the basic characteristics of study participants.

Executive cognition was investigated with widely used cognitive assessment tests. The Go/No-go task was applied to investigate cognitive control, particularly response inhibition control [24, 25]. The Simon’s test was employed to assess attention [26, 27]. The Wisconsin Card Sorting Test (WCST), which primarily measures maintaining set, shifting ability, and feedback utilization, was used to assess cognitive flexibility [28]. Working memory was investigated with the Corsi Block Test [29-31]. The data were supplemented by the results obtained with the Barratt Impulsiveness Scale (BIS-11) [32, 33] and the Emotion Regulation Questionnaire (ERQ) [34].

Clinical anamnestic data on alcohol dependence progression (in years), number of hospitalizations, and remission duration (in days) were also collected.

The data were analyzed using the STATISTICA 12.0 software (StatSoft Europe GmbH). Between-group differences were assessed using the Mann-Whitney U test with a continuity correction. All the mentioned methods were used in accordance with relevant guidelines.

The study was approved by the Ethics Committee of the Mental Health Research Institute (Approval 157/4.2022 on 18^th^ November, 2022) and implemented in compliance with international regulations on research involving human participants: the HRA’s Research Ethics Service, the WMA Declaration of Helsinki (2013), and the Human Rights Act (1998). All the subjects gave written consent to participate in the study and to the subsequent publication of its results in an anonymized form.

## 3 RESULTS

Significant differences in executive cognition indicators, behavioral data, and alcohol dependence anamnestic data were found between groups of indigenous and non-indigenous patients. In particular, indigenous patients with alcohol dependence had more pronounced alterations in the cognitive control domain associated with inhibition errors (p = 0.020), as well as more pronounced alterations in the domain of cognitive flexibility manifested in non-perseveration errors (p = 0.0004), and perseverations (p = 0.0001) (Table **2**). The proportion of errors in the Wisconsin Card Sorting Test was 45% in the indigenous group *versus* 25% in non-indigenous, whereas perseverations in the indigenous group reached 28.3% *versus* 16.7% of those in non-indigenous patients. At the same time, alterations in attention were significantly more pronounced among non-indigenous participants, with 9.47 switching errors compared to 1.33 errors in the indigenous group (p = 0.0002) (Table **2**).

Analysis of behavioral data showed that the indicator of suppression the expression of emotions was higher in the indigenous group (p = 0.028) (Table **2**), which might be a reflection of behavioral norms regarding emotional expression in Asian culture Tuvinians are related to.

Further analysis of alcohol dependence anamnestic data found a significantly higher number of hospitali- zations (p = 0.0005), shortened remission (p = 0.000001), and a higher alcohol dependence progression rate (p = 0.019) in the indigenous group of alcohol-dependent patients (Table **2**).

Next, we performed the k-means cluster analysis of cognitive alteration indicators in indigenous patients with alcohol dependence. The clustering procedure revealed two clusters which had significantly distinct cognitive flexibility indicators. Table **3** summarizes the results of the clustering.

**Fig. (2):**
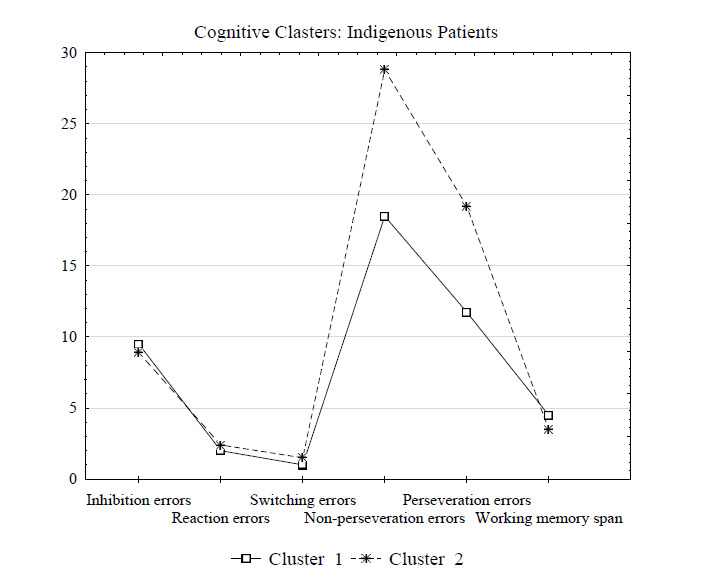
Results of clustering by cognitive alterations indicators based on the k-means in indigenous patients with alcohol dependence.

Cluster 1 included 29 indigenous patients with fewer non-perseveration and perseveration errors (the mean values 18.5 and 11.75, respectively) or mild impairments in cognitive flexibility. Cluster 2 included 45 indigenous patients with a significantly higher number of non-perseveration and perseveration errors (the mean values 28.8 and 19.2, respectively). In addition, patients in Cluster 2 also had a higher number of inhibition errors and a lower working memory span (Fig. **2**); however, the differences in this two indicators were not statistically significant (all p > 0.05). Thus, Cluster 2 consolidated the data of 45 (60.8%) indigenous patients with severe cognitive flexibility impairments.

Fig. (**2**) graphically represents the results of clustering in the group of indigenous patients by cognitive alterations indicators.

Further analysis of anamnestic data showed that indigenous patients from Cluster 2 (with severe cognitive flexibility impairments) had a significantly higher number of hospitalizations (p = 0.040), and their remission was shorter and averaged 90 days *versus* a 180-day remission in patients of Cluster 1 with mild cognitive impairments (p = 0.032) (Table **4**). In addition, indigenous patients from the severe group had greater impulsiveness (p = 0.040), and they were less prone to cognitive reappraisal to regulate their emotions (p = 0.038) (Table **4**).

Subsequently, k-means cluster analysis of cognitive alteration indicators in non-indigenous patients with alcohol dependence revealed that two clusters significantly differed in switching errors, the attention indicator (p = 0.000001) (Table **5**).

**Fig. (3):**
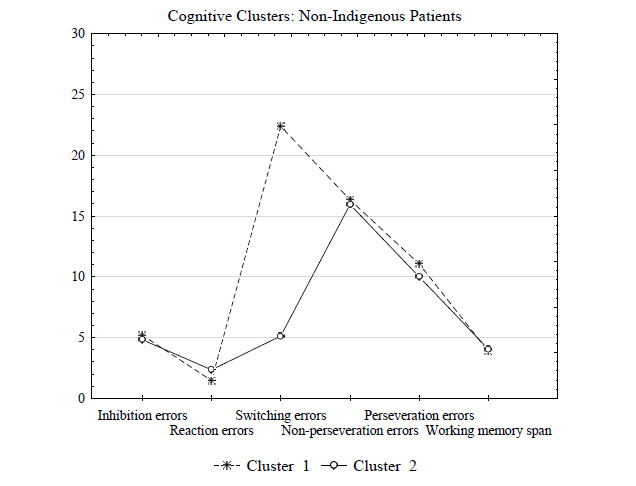
Results of clustering by cognitive alterations indicators based on the k-means in non-indigenous patients with alcohol dependence.

Patients from Cluster 1 (n = 40) had more impaired attention compared to patients from Cluster 2 (n = 52). In particular, switching errors in patients from Cluster 1 were four times higher and amounted to 22.4 *versus* 5.13 in patients from Cluster 2 (Fig. **3**). Thus, Cluster 1 comprised a severe group of non-indigenous patients (40 patients, or 43.5%). Fig. (**3**) graphically represents the results of clustering in the group of non-indigenous patients by cognitive alterations indicators.

Anamnestic data showed that remission in the severe group of non-indigenous patients averaged 135 days compared to the 300-day period of remission in the mild group of non-indigenous patients (Table **4**). Additionally, a severe group of non-indigenous patients with pronounced attention impairment had a higher disease progression rate (p = 0.014) and higher impulsivity (p = 0.042) (Table **4**).

Summarizing the results obtained, severe groups of patients with pronounced cognitive sequelae were found. These groups comprised 60.8% of indigenous patients with an almost two-fold excess of cognitive flexibility errors and 43.5% of non-indigenous patients with more than four times decrease in attention. In severe groups of patients, pronounced cognitive impairments were associated with higher impulsivity, maladaptive emotional coping strategies, frequent hospitalizations (in indigenous patients), and shorter remissions (all p < 0.05).

### 3.1 Clinical Implication

Our study showed that indigenous and non-indigenous patients with severe cognitive alterations had higher disease progression rates, more hospitalizations, and shorter remissions. Based on this evidence and considering existing data on extremely shortened remission in indigenous Tuvinians, we suggested that the approach currently used to treat alcohol dependence may be less effective for some patients, particularly for patients of Tuvinian ethnicity. Therefore, intervention programs should be updated and target vulnerable domains considering the severity of cognitive impairments and remission duration in those patients.

Many suggested that cognitive performance in patients with alcohol dependence can be improved with cognitive training [35, 36]. Cognitive training is a guided and supervised practice structured into a series of neuropsychological exercises or tasks purported to maintain and improve cognitive functions, such as attention, memory, and executive functions. As a type of intervention, cognitive training involves systematic cognitive activation that enhances cognitive function or, if enhancement is not possible, maintains it over time. Studies evaluating the effectiveness of cognitive training programs have confirmed the benefits of this intervention in improving cognition in patients with alcohol dependence [37, 38]. Moreover, there is evidence that cognitive training increases the effectiveness of alcohol dependence treatment since, along with improving cognitive functioning, it contributes to the reduction in the disease symptoms, as well as decreases the risk of relapse [39].

The psychotherapeutic cognitive behavioral intervention has been successfully used to correct maladaptive behavioral patterns, impulsive behavior, and ineffective emotional regulation strategies in patients with alcohol dependence [40]. From the standpoint of a cognitive behavioral approach, addictive behavior is developed under the influence of ineffective coping, a negative self-concept, and a set of expectations from alcohol consumption Then, it leads and leads to a narrowing of the behavioral repertoire, social support destruction, and to depletion of personal and environ-mental coping resources [41, 42]. The development of active coping strategies (problem resolution, search for social support) and effective emotional and behavioral regulation skills helps patients to increase the patient’s involvement in the treatment process, forms an attitude of alcohol abstaining, and reduces the risk of relapse [43].

Given the numerous data on the effectiveness of cognitive training and cognitive behavioral therapy in the treatment of alcohol dependence [38, 41, 44] and based on the evidence on vulnerable cognitive domains obtained in this study, intervention programs for patients of indigenous Tuvinian and non-indigenous ethnicity should be designed and widely implemented. Tailored intervention must target cognitive alterations in the executive cognition domain, as well as impulsivity and emotional regulation skills. In addition, for Tuvinian patients, culture-based intervention must be provided to advance resilience in this group of indigenous people of Siberia.

## 4 DISCUSSION

According to the available data, 30 to 50% of patients with alcohol dependence are readmitted to the hospital within one year [19, 20], which is confirmed by the data obtained in this study on a shorter duration of remission and a greater number of hospitalizations among the indigenous Tuvinian patients and almost half of non-indigenous patients with alcohol dependence. In addition, the average remission in indigenous patients was significantly shorter than that in the group of non-indigenous patients, which indicates greater severity of alcohol-related health consequences in the indigenous people and confirms the existing world data [45-47]. On the other hand, the approaches currently used for alcohol dependence treatment may be less effective for patients of the indigenous Tuvinian ethnicity.

Large evidence links the inability to abstain from alcohol consumption and maintain a long-term remission with impaired or weakened cognitive functions that regulate behavior, particularly executive functions [9, 10, 14, 48, 49]. In our study, both non-indigenous and indigenous Tuvinian patients (the latter with shorter remissions) showed a significantly higher number of switching errors when performing sorting in the Wisconsin Card Sorting Test (60.8% of indigenous patients) and significantly more attention errors in the Simon’s test (43.5% of non-indigenous patients). These data indicate that cognitive flexibility and attention were vulnerable cognitive domains in alcohol-dependent patients of indigenous and non-indigenous ethnicity. These domains and, particularly, their key severity indicators (perseveration errors, non-perseveration errors, and switching errors) require special attention when planning intervention. Since neurocognitive indicators are increasingly considered important transdiagnostic targets for alcohol and substance use disorder treatment [50, 51], intervention programs must be updated, and cognitive training targeted at key severity indicators should be included. In addition, since patients in all severe groups had significantly higher impulsivity and were less committed to adaptive emotional regulation strategies, cognitive behavioral therapy should be implemented for basic intervention.

## CONCLUSION

While some limitations of the study exist, including a male sample, whose data were collected to homogenize potential sex-related effects of alcohol on cognition, our findings are highly relevant for improving intervention in both indigenous and non-indigenous patients with alcohol dependence. Considering the high prevalence of alcohol dependence among the indigenous population in different parts of the world [21, 45, 47], as well as increased mental health consequences due to the recent pandemic [52-55], the study results contribute to existing approaches to personalized treatment of alcohol dependence among patients of the indigenous Tuvinian ethnicity.

## Figures and Tables

**Fig. (1):**
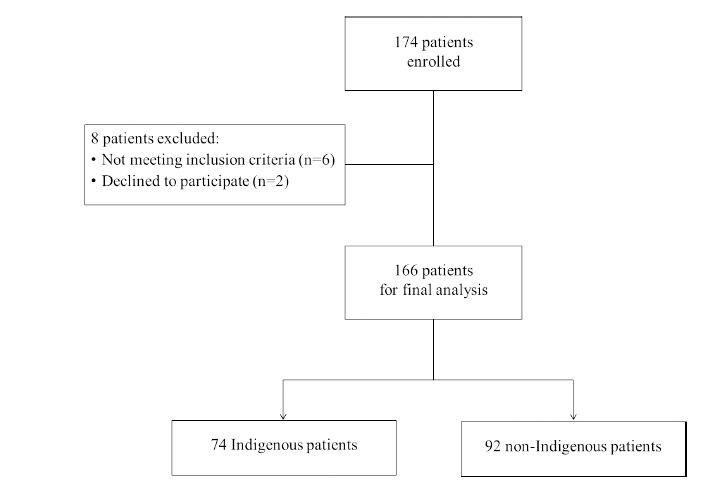
Flowchart of the study sample.

**Table 1:** Basic characteristics of the study participants.

Indicator	Indigenous Patients	Non-indigenous Patients
Participants, n	74	92
Age, years	43.85 ± 7.46	44.92 ± 7.82
Education, years	12.9 ± 3.1	13.7 ± 2.9
Alcohol dependence duration, years	10.34 ± 7.06	11.02 ± 6.76

**Table 2:** Executive cognition indicators, behavioral data, and alcohol dependence anamnestic data of participants (significant differences based on the mann–whitney u test with a continuity correction are shown in bold).

Indicator	Indigenous Patients	Non-indigenous Patients
Executive cognition		
**Cognitive control: Inhibition errors (Go/No-go)**	**11.06 ± 8.59**	**6.09 ± 5.52**
Cognitive control: Reaction errors (Go/No-go)	2.11 ± 1.64	2.16 ± 1.47
**Attention: Switching errors (Simon’s Test)**	**1.33 ± 1.5**	**9.47 ± 8.56**
**Cognitive flexibility: Non-perseveration errors (WCST)**	**24.87 ± 7.08**	**17.26 ± 7.07**
**Cognitive flexibility: Perseveration errors (WCST)**	**16.40 ± 4.79**	**10.76 ± 3.92**
Working memory: Working memory span (Corsi Block Test)	3.81 ± 2.01	4.32 ± 1.55
Behavioral data		
Impulsivity (BIS-11)	63.67 ± 9.69	62.19 ± 7.03
Emotional regulation: Cognitive reappraisal (ERQ)	31.96 ± 5.74	29.65 ± 5.14
**Emotional regulation: Expression suppression (ERQ)**	**20.79 ± 4.27**	**17.7 ± 4.92**
Alcohol dependence anamnestic data		
**Hospitalizations**	**2.84 ± 3.8**	**1.4 ± 1.69**
**Last remission, days**	**206.64 ± 596.09**	**549.02 ± 652.67**
**Alcohol dependence progression, years**	**4.84 ± 3.88**	**8.4 ± 7.96**

**Table 3:** Cluster analysis results in indigenous patients with alcohol dependence based on the k-means cluster analysis (significant differences are shown in bold).

Cognitive Domain	Indicator	Cluster 1 (n = 29)	Cluster 2 (n = 45)	F	p-value
Cognitive control	Inhibition errors	9.5	8.9	0.023	0.881
	Reaction errors	2.0	2.4	0.136	0.719
Attention	Switching errors	1.0	1.5	0.249	0.627
**Cognitive flexibility**	**Non-perseveration errors**	**18.5**	**28.8**	**18.886**	**0.001**
	**Perseveration errors**	**11.75**	**19.2**	**27.841**	**0.0002**
Working memory	Working memory span	4.5	3.5	0.618	0.447

**Table 4:** Differences in behavioral and anamnestic data between severe and mild groups of indigenous and non-indigenous patients with alcohol dependence based on the Mann–Whitney U test with a continuity correction (significant differences between severe and mild groups are shown in bold).

Indicator	Indigenous Patients		p-value	Non-indigenous Patients		p-value
	Cluster 1 (Mild Group) n = 29	Cluster 2 (Severe Group) n = 45		Cluster 1 (Severe Group) n = 40	Cluster 2 (Mild Group) n = 52	
**Impulsivity (BIS-11)**	**61.0**	**65.0**	**0.040**	**66.0**	**63.0**	**0.042**
**Emotional regulation: Cognitive reappraisal (ERQ)**	**33.0**	**29.5**	**0.038**	29.5	31.0	0.151
**Emotional regulation: Expression suppression (ERQ)**	22.0	21.0	0.448	**14.0**	**22.0**	**0.029**
Alcohol dependence duration, years	9.5	7.0	0.527	9.0	9.0	0.542
**Hospitalizations**	**1.0**	**2.5**	**0.040**	1.0	1.0	0.499
**Latest remission, days**	**180.0**	**90.0**	**0.032**	**135.0**	**300.0**	**0.027**
**Alcohol dependence progression, years**	6.5	5.0	0.054	**4.5**	**6.0**	**0.014**

**Table 5:** Cluster analysis results in non-indigenous patients with alcohol dependence based on the k-means cluster analysis (significant differences are shown in bold).

Cognitive Domain	Indicator	Cluster 1 (n = 40)	Cluster 2 (n = 52)	F	P-value
Cognitive control	Inhibition errors	5.2	4.83	0.079	0.781
	Reaction errors	1.5	2.4	2.618	0.114
**Attention**	**Switching errors**	**22.4**	**5.13**	**117.13**	**0.000001**
Cognitive flexibility	Non-perseveration errors	16.3	15.96	0.040	0.843
	Perseveration errors	11.1	10.0	0.780	0.383
Working memory	Working memory span	3.87	4.04	0.100	0.754

## Data Availability

The data supporting the findings of the article is not freely available due to ethical restrictions.

## LIST OF ABBREVIATIONS

WCST: Wisconsin Card Sorting Test
ERQ: Emotion Regulation Questionnaire

## STANDARDS OF REPORTING

STROBE guidelines were followed.
